# Internal Fixation for Unstable Distal Ulnar Fractures by 2.7 mm Semitubular Hook Plate

**DOI:** 10.1155/aort/5663025

**Published:** 2024-12-24

**Authors:** Mohamed I. Abulsoud, Mohammed Elmarghany, Ahmed R. Zakaria, Ehab A. Alshal, Mohamed Moawad, Ehab A. Elzahed, Mohamed F. Elhalawany, Bahaa A. Kornah

**Affiliations:** ^1^Department of Orthopedic Surgery, Faculty of Medicine, Al-Azhar University, Cairo, Egypt; ^2^Department of Orthopedic Surgery, Faculty of Medicine, Helwan University, Cairo, Egypt

**Keywords:** distal ulnar fractures, hook plate, ulnar head level of evidence level IV, wrist fractures

## Abstract

**Objective:** The purpose of this study is to investigate the outcomes of the use of a 2.7 mm semitubular hook plate for internal fixation of unstable metaphyseal ulnar fractures.

**Methods:** Between January 2015 and July 2019, 30 consecutive patients with a recent unstable distal ulnar fracture were included in this prospective case series. All patients were subjected to follow-up with the time of union, range of motion, pain using a Visual Analog Scale (VAS), and radiological and functional outcome using the quick Disabilities of the Arm, Shoulder, and Hand (DASH) score and Mayo wrist score after 12 months.

**Results:** The mean age of the patients was 45.3 ± 10 years. There were 18 males (60%) and 12 females (40%), and there were 16 patients associated with distal radius fractures (53.33%). According to the AO classification of distal ulnar fractures, 3 fractures were type A2.1 (10%), 9 were type A2.2 (30%), 8 fractures were type A2.3 (26.67%), and 10 fractures were type A3 (33.33%). All fractures have been united with a mean duration of 9 ± 1.4 weeks, the mean supination was 81.4° ± 3.5°, the mean pronation was 81.3° ± 4.5°, the mean flexion was = 71.7° ± 3.6°, and the mean extension was = 81.7° ± 3.4°. The mean VAS was 1.1 ± 1 points, the mean DASH score was 9.3 ± 5.6 points, and the mean Mayo wrist score was 88.5 ± 7.2 points; 17 patients were excellent (56.67%) and 10 patients were good (33.33%) while 3 patients had satisfactory outcome (10%).

**Conclusion:** Using the 2.7 mm semitubular hook plate is a successful choice for internal fixation of unstable distal ulnar fractures isolated or associated with distal radius fractures with a favorable union time, functional outcome, and range of motion with minimal complications.

**Summary**

• The surgical management of distal ulnar fractures is challenging and controversial.
• The study evaluated the use of a conventional 2.7 mm hook plate as an economic and available treatment choice.
• The functional outcome was favorable in 90% of patients with minimal complications.

## 1. Introduction

The metaphyseal distal ulnar fracture is the fracture that lies within 5 cm from the dome of the ulnar head [1], the distal ulnar fractures are relatively uncommon and usually involve the ulnar styloid [2] unlike styloid fractures, the incidence of associated distal radius fractures with metaphyseal ulnar fractures is 5%–6% [3], typically in elderly patients with osteoporotic fractures [4], and isolated fractures could occur due to direct trauma to the ulnar side of the wrist [5].

The distal ulna is a fixed point around which the hand and radius rotate in various activities of daily living [6], the ulnar head is a keystone structure in maintaining the stability of the distal radioulnar joint (DRUJ) and the triangular fibrocartilage complex (TFCC) of the wrist [7], and improper treatment of the distal ulnar fractures could lead to limited forearm rotation, persistent pain, DRUJ instability, and arthritis [1].

According to the OTA/AO classification [8], distal ulnar fractures (2U3A) are classified into type A1 which represents the ulnar styloid fractures and type A2 which represents extra-articular fractures and subclassified into 2U3A2.1, 2U3A2.2, and 2U3A2.3 which are the spiral, oblique, and transverse fractures, respectively, while type A3 is the multifragmentary fracture.

Unlike the distal radius, the distal ulnar fractures are usually not well appreciated and often result in inadequate treatment [9], the distal ulnar fracture may require fixation either as an isolated injury or in combination with distal radius fractures, angulation more than 10°, more than 3 mm ulnar variance, or translation more than one-third of the diameter of the ulna are the main indications for fixation, and such criteria should be considered in cases of intact or anatomically reduced distal radius [10, 11].

Several options for fixation have been described, such as tension band wiring [12], minicondylar blade plate [10], locking plate [11], and headless compression screws [13]; the use of anatomical distal ulnar hook plate gained a lot of popularity in the last few years [14, 15].

However, no fixation option is superior to others [16], and the cost and availability of such options are still an issue that invites a more economical, stable, and available fixation option.

The purpose of this study is to investigate the outcomes of the use of a 2.7 mm semitubular hook plate for internal fixation of unstable metaphyseal ulnar fractures.

## 2. Methods

This prospective case series included 30 consecutive patients with a recent unstable distal ulnar fracture between January 2015 and July 2019.

The inclusion criteria included adult populations with a closed unstable distal ulnar fracture either associated with fracture distal radius or isolated injury, and the fracture is considered unstable if there is angulation more than 10° in any plane, displacement of the distal fragment of more than one-third of the ulnar diameter, more than 3 mm of ulnar variance, or highly comminuted fracture.

The exclusion criteria included nondisplaced or reduced fractures after fixation of the distal radius fracture, styloid ulnar fractures, pathological fractures, open fractures, patients with peripheral vascular diseases, and a population less than 18 years old.

All cases were subjected to open reduction and internal fixation by a 2.7 mm semitubular hook plate which is fixed distally by two screws and 3-4 screws proximally using bicortical 2.7 mm screws.

Preoperative planning included history taking and thorough clinical examination including neurovascular examination, radiographic evaluation using plane X-ray at 2 different views including wrist (posteroanterior and lateral views) and elbow (anteroposterior and lateral views), and computerized tomography in cases of associated distal radius fracture as well as routine preoperative investigations to confirm the fitness of the patients for anesthesia.

Ethical approval was obtained from the institutional ethics committee and informed consent forms from all patients were received.

### 2.1. Surgical Technique

All cases have been performed under general anesthesia with the use of a tourniquet. The fixation of distal radius fractures is performed first in cases of associated injuries and confirms that the distal ulnar fractures are still unstable. Seven cases have been fixed by percutaneous pinning with K-wires, and 9 cases were fixed by volar locked plates.

**Figure 1:**
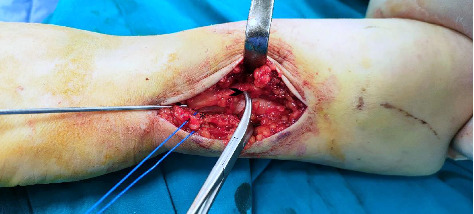
The surgical approach of fixation for distal ulnar fracture. The sign ⁣^∗^ refers to the dorsal sensory branch of the ulnar nerve, and the arrowhead refers to the extensor carpi ulnaris tendon which is usually found so close to the distal ulnar metaphysis; a preliminary *k* wire with the aid of bone-holding forceps was used to maintain the reduction while preparing and inserting the plate.

A longitudinal skin incision was made on the ulnar border of the distal forearm, the subcutaneous tissue has been dissected carefully to identify and protect the dorsal sensory branch of the ulnar nerve which is seen about one inch from the ulnar styloid. In cases of multifragmentary fractures, care should be taken to preserve the soft tissue envelope to preserve the blood supply of the small fragments and the soft tissue relationship of the bone, once proper reduction is achieved by direct manual light traction or the use of small bone levers, a preliminary k-wire could be used to maintain the achieved reduction (Figure 1).

**Figure 2:**
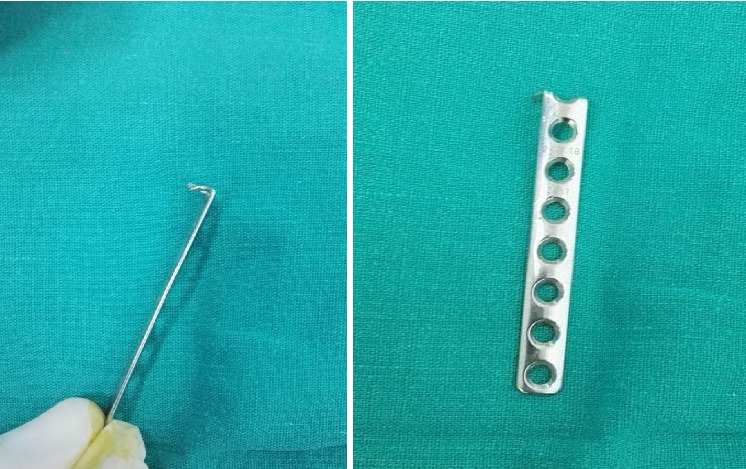
The 2.7 mm semitubular plate after prebending its tip to make a hook.

After exposure to the fracture site and proper reduction, a 2.7 mm semitubular plate is used after cutting its upper edge and bending both pillars to make them like a hook (Figure 2).

**Figure 3:**
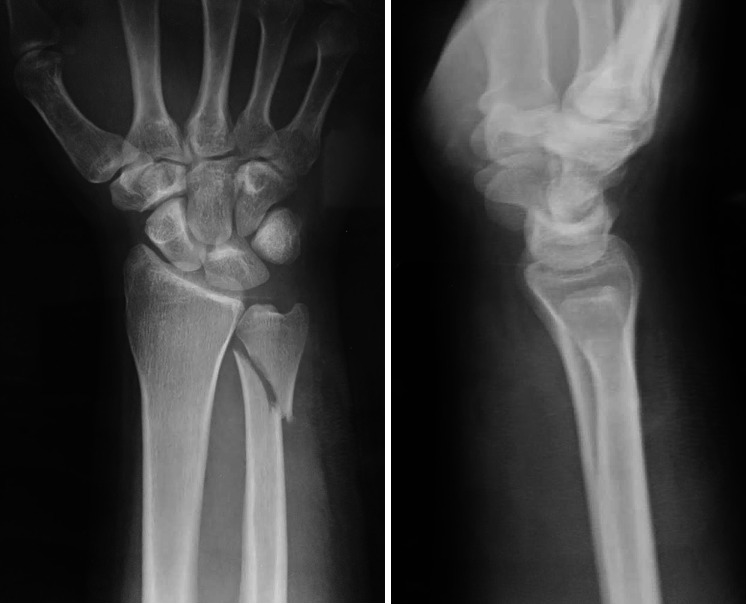
X-ray wrist anteroposterior and lateral view of a 48-year-old male patient with unstable distal ulnar fracture.

**Figure 4:**
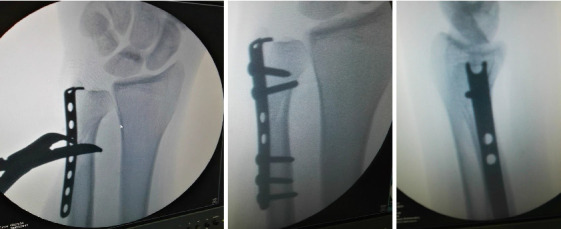
Fluoroscopic photos show the application of the 2.7 mm semitubular hook plate and the final images after fixation.

**Figure 5:**
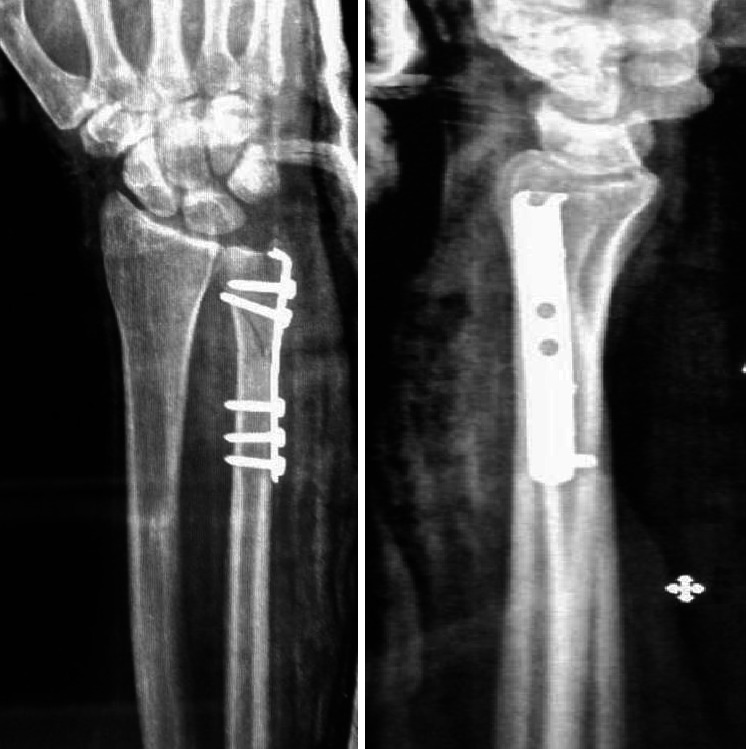
Postoperative X-ray wrist anteroposterior and lateral views show the fracture after fixation by a 2.7 mm semitubular plate.

The plate should be applied on the dorsoulnar surface, and its hook should be located embarrassing the ulnar styloid to not jeopardize the TFCC, some proximally directed force should be applied to engage the hook into the ulnar head, then fixation of the fracture by 2.7 mm screws to conclude the fixation (Figures 3, 4, and 5).

Postoperatively, an arm splint is applied for 3 weeks, followed by a removable splint for another 3 weeks, with emphasis on mobilizing the fingers and then the wrist.

**Figure 6:**
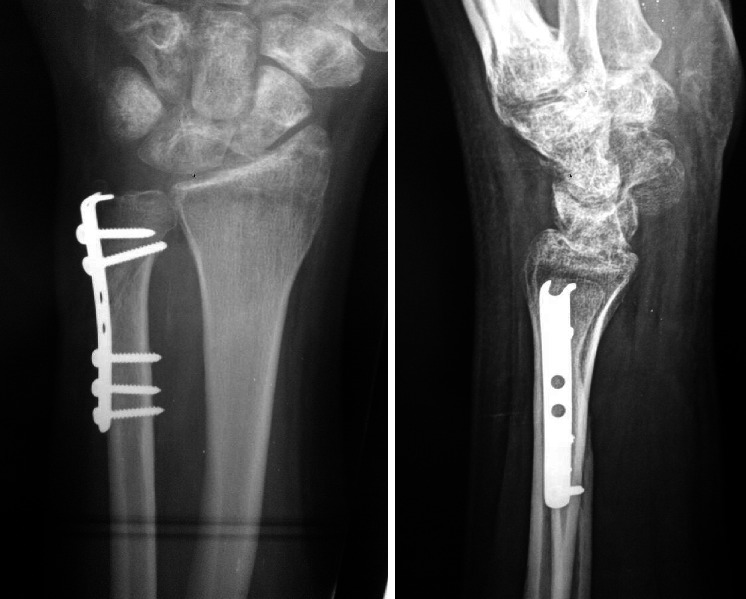
X-ray wrist anteroposterior and lateral views show follow-up of the patient after union.

Afterward, the patients were encouraged to mobilize the wrist and to do passive rather than active pronation and supination (Figure 6).

### 2.2. Outcome Measures

All patients were submitted to follow-up after 12 months postoperatively with the time of union, range of motion (flexion, extension supination, and pronation of the wrist), pain using the Visual Analog Scale (VAS), and functional outcome using the quick Disabilities of the Arm, Shoulder, and Hand (DASH) score [17] and Mayo wrist score [18].

Radiographic evaluation for preoperative and last follow-up postoperative wrist posteroanterior X-ray has been evaluated for measuring radial height, radial inclination, and ulnar variance.

Radial inclination was measured on a posteroanterior X-ray by measuring the angle formed between the long axis of the radius and a line drawn from the distal tip of the radial styloid to the ulnar corner of the lunate fossa. Radial height is measured by finding the long axis of the radius and extending a line perpendicular to it at the tip of the radial styloid on a posteroanterior radiograph. The distance between this line and the distal-most point of the ulnar dome was recorded. The ulnar variance was measured on a posteroanterior radiograph using the method of perpendiculars [19].

### 2.3. Statistical Analysis

Data were analyzed using Statistical Program for Social Science (SPSS), Version 15.0 (SPSS Inc., Chicago, Illinois). Quantitative data were expressed as mean ± standard deviations after confirmation of normal distribution. Data that were not distributed normally were expressed as medians and interquartile ranges. Qualitative data were expressed as frequencies and percentages. *p* value < 0.05 was statistically significant. A *t*-test was performed to study the association of the demographic data (age, associated distal radius, and the fracture type according to AO classification).

## 3. Results

The study included thirty patients. The mean age was 45.3 ± 10 (range 29–61) years. There were eighteen males (60%) and twelve females (40%), and there were 16 patients associated with distal radius fractures (53.33%). Among the distal radius fracture cases, 9 of them were type C2 (56.25%), 4 cases were A3 (25%), and 3 patients presented with type C1 fractures (18.75%).

According to the AO classification of distal ulnar fractures, three fractures were type A2.1 (10%), 9 were type A2.2 (30%), 8 fractures were type A2.3 (26.67%), and 10 fractures were type A3 (33.33%) (Table 1).

All fractures have been united with a mean duration of 9 ± 1.4 (range 7–12) weeks; as regards the range of motion, the mean supination was 81.4 ± 3.5 (range 75–88) degrees, the mean pronation was 81.3 ± 4.5 (range 70–88) degrees, the mean flexion was 71.7 ± 3.6 (range 65–78) degrees, and the mean extension was 81.7 ± 3.4 (range 75–88) degrees; there were no cases of residual DRUJ instability at the end of the follow-up. The mean VAS was 1.1 ± 1 points (range 0–3).

When measuring the functional outcome after 12 months postoperatively, the mean quick DASH score was 9.3 ± 5.6 points (range 0–20.5); according to the Mayo wrist score, the mean score was 88.5 ± 7.2 points (range 75–100); 17 patients were excellent (56.67%), 10 patients were good (33.33%), and 3 patients had satisfactory outcomes (10%) (Table 2).

As regards the radiographic evaluation of wrist parameters, the mean radial height improved from a mean of 10.13 ± 2.8 mm (range 3–14) preoperatively to 12.2 ± 0.96 mm (range (10–14) postoperatively which is statistically significant (*p*=0.04), and the mean radial inclination improved from 17.6 ± 7.18° (range 3–25) preoperatively to 23.1 ± 1.95° (range 20–27) postoperatively (*p*=0.87), while the ulna variance was +0.8 ± 1.86 mm (range −4 to +2.3) in preoperative assessment and +0.36 ± 0.96 mm (range −2 to +1.7) postoperatively (*p*=0.3) (Table 3).

  Subgroup analysis: When analyzing the correlation between age, fracture type, and associated distal radius fractures and the functional outcomes, there was no statistically significant correlation (*p* value was ≤ 0.05) in all tests.
  Complications: The reported complications were superficial wound infection in 2 cases (6.67%), one of each was in each group, both improved with local wound care and broad-spectrum antibiotics at 3 weeks postoperatively, there were 3 patients (10%) with limited wrist flexion with minor limitation in the functional activity of daily living, and one patient reported hardware prominence with crepitus which required plate removal 10 months postoperatively after confirming the bony union (Table 4).

## 4. Discussion

Metaphyseal distal ulnar fracture fixation is challenging due to the short segment of fixation, associated comminution, the presence of osteoporosis, and thin, soft tissue envelope, besides the triangular shape of the distal ulna makes the placement of the plate is poorly tolerated by the patients. The volar placement of plates is more convenient, but it carries a considerable risk of injury to the ulnar nerve and vessels [1, 5, 10].

Fractures of distal ulna associated with distal radius fractures are usually well-tolerated making the fixation not necessary in all cases [20, 21]; however, displaced and unstable fractures if neglected will lead to devastating complications such as longitudinal forearm instability, DRUJ instability, and arthritis [3, 5, 22].

In the literature, the fixation of metaphyseal ulnar fractures has been achieved by different methods, percutaneous pinning carries the risk of loosening and pin tract infection [23], tension band wiring cannot be utilized in comminuted fractures, and implant-related complications are not uncommon [12]. The use of headless compression screws has been reported to be used in intraarticular fractures either as isolated injury [24, 25] or associated with a distal radius fracture [26]; recently, the use of headless compression screws has been expanded to include metaphyseal fractures as an intramedullary screw by Oh and Park [13], the study included 11 patients with a mean follow-up of 26.6 weeks (about 6 months), the mean union time was 6.5 weeks, the mean quick DASH score was 14.6, and mean VAS score was 1.09. However, the age group was high (70 years and all were women), suggesting low functional demand, and the rotation in comminuted cases is still a concern.

The use of anatomical locked distal ulnar plates gained popularity in the last few years. Lee et al. [14] evaluated the results of distal ulnar LCP in 25 patients with a mean age of 62.3 years, all patients achieved bony union in a mean of 12.5 weeks with a good range of motion and the average modified Mayo wrist score was 87 points, and average quick DASH score was 14 points.

In the retrospective study by Han, Hong, and Kim [15], 17 patients were included in this study with a mean age of 58.9 years, all of them were associated with distal radius fracture and the distal ulna was fixed by distal ulnar LCP, the average follow-up was 15 months, all fractures had a bony union with an average of 11.7 weeks with a very good range of motion and radiographic measurement, the mean DASH score was 11 points, and 6 patients gave excellently and 11 patients gave good results according to modified Sarmiento's score of the wrist. Meluzinová et al. [27] used the same plate in eighteen patients, all of them associated with distal radius fractures with a mean age of 58 years, with a follow-up of 9 months, the average Mayo wrist score was 84 points, and the quick DASH score was 7.4 points with a good postoperative range of motion.

Recently, Gauthier et al. [28] retrospectively evaluated the outcome of 48 patients with combined distal ulnar fractures with distal radius fractures, the distal ulnar fractures were fixed by anatomical hook plate distal ulna LCP, the follow-up time was 28 months, and the functional outcomes of the patients were very good to excellent as regards Q-DASH score, Mayo wrist score, and range of motion; however, high complication rate was observed (45%), and the most common was pain and discomfort requiring plate removal in 14 patients (29%).

Stock et al. [29] investigated a different design of anatomical LCP which is applied volarly, and without a hock in the ulnar head; they included 20 patients with a mean age of 70 years old, and the mean DASH score, the PRWE score, and the VAS after one year had no significant difference to the uninjured side (Table 5).

The sample size was comparable to the published articles in the same area, this could be explained by the fact that many cases with distal ulnar fractures are stable, especially after the reduction and fixation of distal radius fractures, and the follow-up period (12 months) is also comparable to the referenced articles. The mean age of patients in this study is younger than that in other studies, this could be why the time for the union was relatively shorter than that in other studies, the functional outcomes in this study were comparable in all studies, and some variables were found similar (Mayo wrist score), or variable as VAS or Q-DASH score, and this could be due to the nature of the outcome itself; however, all results were relatively similar as regards the functional outcome, and the rate of complications was higher in anatomical LCP and minicondylar plate (Table 5).

However, the cost and availability of the distal ulnar LCP may limit its use in all cases, and still, a high rate of complications was observed; some modifications are ongoing to have a lower profile plate and to minimize soft tissue irritation [29].

The immobilization method and time have been studied in the literature (Table 5). The immobilization method is usually a short arm splint, the immobilization time shows gross variability from 2 to 6 weeks postoperatively, the immobilization time depends on the bone quality, the degree of comminution of the fracture, the stability of the fixation, and patient compliance, it is recommended to use short arm splint rather than long arm one, and for a minimum until stitch removal, a removable splint could be used during rehabilitation time.

The use of hook plate has been utilized in different areas in the skeleton such as ankle fractures, olecranon, the base of the fifth metatarsal, and radial styloid. Heim and Niederhauser described the use of a 3.5 mm one-third semitubular plate successfully in the management of distal ulnar fractures; however, the use of 3.5 mm plates may lead to inadequate fixation of the distal fragment in several types of distal ulnar fractures [30].

In this study, the use of 2.7 mm semitubular plates for fixation of distal ulnar fractures was not mentioned before in the literature, the one-quarter tubular plate is a low profile plate of 1 mm with a diameter of 7 mm and a hole spacing of 8 mm [31] which allows fixation of the short distal fragment by 2 screws as a minimum, plus the hook which adds more stability to the construct when anchors the ulnar head, so this plate does not lead to more soft tissue disruption or hardware prominence, in this study, only one patient needed to remove hardware, when analyzing his radiograph, it is found that the plate is dorsal rather than a dorsoulnar site which is the recommended for plate placement to avoid interference with the function of Extensor Carpi Ulnaris tendon. The use of minicondylar blade plate for fixation of distal ulnar fractures was associated with hardware prominence in seven out of twenty-four patients (29.16%) [10].

The subanalysis using a t-test found a nonstatistically significant difference between isolated cases and cases with associated distal radius fractures, to avoid overdone surgeries, it is emphasized that not all associated ulnar fractures after fixation of distal radius fractures need fixation and most of them became stable which does not require further treatment.

Once the distal radius fracture was reduced and fixed, the stability of the distal ulnar fracture was evaluated, and the reduction and fixation of distal radius fracture if done properly restored the radiological parameters of the wrist (Table 4); to minimize bias, subgroup analysis has been done which found no differences between both groups, the radiological parameters of the wrist have been evaluated for all cases, and the postoperative wrist parameters are comparable to the normal ranges which emphasize the recommendation to pay more attention for the reduction and fixation of distal radial fractures in the context of distal forearm fractures.

There were no cases of residual DRUJ instability. This could be explained by the fact that anatomical restoration of the bony relations between the sigmoid notch of the distal radius and the ulnar head usually leads to excellent outcomes with distal radius reduction and fixation with or without fixation of the ulna [14, 23, 32]. Even when DRUJ stability has been evaluated by CT scan by earlier studies [14, 27], no residual instability has been encountered; this means that the clinical intraoperative evaluation is the most important parameter to detect and address such injury.

Despite the novel use of the 2.7 semitubular plates in treating unstable distal ulnar fractures and its success, the study carries some limitations: the lack of a control group, especially in comparison to the anatomical distal ulnar LCP, the cost-effectiveness of both implants could be studied as well as the use of both plates in the older age group with more osteoporotic fractures.

### 4.1. Conclusion

The use of the 2.7 mm semitubular hook plate is a successful choice for internal fixation of unstable distal ulnar fractures either isolated or associated with distal radius fractures with a favorable union time and functional outcome and range of motion with minimal complications. The use of such a plate is a suitable alternative to anatomical LCP with more availability and less cost with a comparable outcome.

## Figures and Tables

**Table 1:** Demographic data.

Characteristics	Value
Age (years)	
Minimum	29
Maximum	61
Mean (SD)	45.3 (10)
Gender	
Male	18
Female	12
Distal radius fracture	
Associated	16 (53.33%)
Isolated	14 (46.67%)
AO classification of distal radius cases	
Type C2	9 (56.25%)
Type A3	4 (25%)
Type C1	3 (18.75%)
Fracture type	
A2.1	3 (10%)
A2.2	9 (30%)
A2.3	8 (26.67%)
A3	10 (33.33%)

**Table 2:** Clinical results.

Characteristics	Value
Time to union (weeks)	
Minimum	7
Maximum	12
Mean (SD)	9 ± 1.4
Range of motion	
Supination	81.4° ± 3.5 (range 75°–88°)
Pronation	81.3° ± 4.5 (range 70°–88°)
Flexion	71.7° ± 3.6 (range 65°–78°)
Extension	81.7° ± 3.4 (range 75°–88°)
Visual Analog Scale	
Minimum	0
Maximum	3
Mean (SD)	1.1 (1)
Quick DASH score	
Minimum	0
Maximum	20.5
Mean (SD)	9.3 (5.6)
Mayo wrist score	
Minimum	75
Maximum	100
Mean (SD)	88.5 (7.2)

**Table 3:** Radiographic evaluation preoperatively and at 12 months postoperatively.

	Preoperative			12 months postoperative			*p* value
	Mean	SD	Range	Mean	SD	Range	
Radial height	10.13 mm	2.8	3–14	12.2 mm	0.96	10–14	0.04^∗^
Radial inclination	17.6°	7.18	3–25	23.1°	±1.95	20–27	0.87
Ulnar variance	+0.8 mm	±1.86	−4–+2.3	+0.36 mm	±0.96	−2–+1.7	0.3

^∗^Statistically significant.

**Table 4:** Subgroup analysis of functional and radiographic outcomes of both groups.

Outcome measures	Without distal radius fracture (*n* = 14)	With distal radius fracture (*n* = 16)	Statistical correlation (*p* value)
Union time (weeks)	8.6 ± 1.2 (range 7–11)	9.3 ± 1.5 (range 7–12)	0.15 (NS)
Range of motion (degrees)			
Supination	82.1 ± 3.2 (range 75–88)	80.7 ± 3.8 (range 75–85)	0.56 (NS)
Pronation	82.2 ± 4.3 (range 75–88)	80.4 ± 4.6 (range 70–88)	0.48 (NS)
Flexion	72.5 ± 3.1 (range 67–77)	70.9 ± 3.9 (range 65–78)	0.36 (NS)
Extension	83.0 ± 3.4 (range 75–88)	80.5 ± 3.1 (range 75–85)	0.30 (NS)
Pain (VAS score)	0.9 ± 1.0 (range 0–2)	1.3 ± 1.1 (range 0–3)	0.34 (NS)
Quick DASH score	8.1 ± 5.0 (range 0–18.5)	10.4 ± 6.1 (range 0–20.5)	0.41 (NS)
Mayo wrist score	90.3 ± 6.5 (range 80–100)	86.8 ± 7.6 (range 75–98)	0.31 (NS)
Radial height (mm)	11.8 ± 1.1 (range 10–13)	12.4 ± 0.9 (range 10–14)	0.20 (NS)
Radial inclination (°)	23.3 ± 1.8 (range 20–26)	22.9 ± 2.2 (range 20–25)	0.57 (NS)
Ulnar variance (mm)	0.22 ± 0.73 (range −1.8 to +1.6)	0.48 ± 1.08 (range −2.0 to +2.3)	0.33 (NS)
Preoperative ulnar variance (mm)	−0.25 ± 1.1 (range −3.5 to +1.2)	−0.30 ± 0.85 (range −2.8 to +1.0)	0.82 (NS)
Complications			
Superficial infection (*n*, %)	1 (7.1%)	1 (6.25%)	1.0 (NS)
Hardware prominence (*n*, %)	0 (0%)	1 (6.25%)	0.56 (NS)
Wrist flexion limitation (*n*, %)	0 (0%)	3 (18.75%)	0.23 (NS)
Hardware removal (*n*, %)	0 (0%)	1 (6.25%)	0.56 (NS)
Residual DRUJ instability (*n*, %)	0 (0%)	0 (0%)	—

**Table 5:** Comparison of the data of the articles in the literature as compared to the data of this study.

The study	Implant used	Immobilization	Sample size	Mean age (years)	Follow-up period (month)	Mean time to union (weeks)	Mean VAS	Mean Q-DASH	Mean Mayo wrist score	Other
This study	2.7 mm semitubular plate	3 weeks short arm splint followed by 3 weeks in a removable splint	30	45.3	12	9	1.1	9.3	88.5	

Oh and Park [13]	Intramedullary headless screw	2 weeks in a short arm splint	11	70	6	6.5	1.06	14.6	—	

Lee et al. [14]	Anatomical LCP	4-5 weeks, sugar-tong splint for 7–10 days, then a removable splint	25	62.3	N/A	12.5	N/A	14	87	

Han, Hong, and Kim [15]	Anatomical LCP	Short arm splint, time unavailable	17	58.9	15	11.7	N/A	11		Modified Sarmiento's score Excellent 6 Good 11 Fair 0 Poor 0

Meluzinová et al. [27]	Anatomical LCP	N/A	18	58	9	N/A	N/A	7.4	84	

Gauthier et al. [28]	Anatomical LCP	Short arm cast, 6 weeks	48	63	28	N/A	0.6	12	90	

Stock et al. [29]	Volar anatomical LCP	Forearm splint, 10–14 days	20	70	14	12 (95%) 24 (100%)	1.2	15.9	N/A	PRWE (Patient Rated Wrist Evaluation) 14.5

Ring et al. [10]	Minicondylar blade plate	Volar splint for 2–6 weeks	24	51.7	26	N/A	N/A	N/A	N/A	Modified Sarmiento's score Excellent 6 Good 15 Fair 4

Abbreviations: DASH = Disabilities of the Arm, Shoulder, and Hand; N/A = not available; VAS = Visual Analog Scale.

## Data Availability

The datasets used and analyzed during the current study are available from the corresponding author upon request.
